# Editorial Notes

**Published:** 1910-09

**Authors:** 


### Editorial Notes.

**British Medical Association.**

THE seventy-eighth annual meeting, which coincided with the seventh birthday of the Representative Body, had many proofs before it of the success and vigour with which the Association is carried on under the present constitution. The attempt to obtain a Royal Charter, indeed, has been formally given up, but many of the objects sought for will be attained just as well by an amended Memorandum of Association under the Companies' Act. Meanwhile there is rapid growth both of the membership and of the work in various directions undertaken for the benefit of the general practitioner. The publication of the analyses of "Secret Remedies" has been a great success. The public has been widely interested and surprised by the revelations. 42,000 copies have been sold, and 20,000 more already ordered.

It was decided not to give a scanty Accident Insurance with the *Journal*, but a committee has arranged favourable terms for a really comprehensive one for members desiring it. A similar decision was given against spending money on a Central Research Laboratory, in view of the many existing laboratories, such as those of Dr. Mond's bequest, which are badly filled, but the Association continues to support and organise scientific research, and was able in particular to bring forward a most valuable report on the mininal dose of chloroform which is adequate without causing danger to life. Such burning questions as the dangers of School Athletics, the regulation of District Nursing Associations, ill-paid lectures on First Aid and Hygiene, and the prevention of Ophthalmia Neonatorum have been taken in hand, and carefully considered rules have been issued to check the evils complained of. Among many other subjects on which much work has been expended is the reform of death registration and of the coroner's jurisdiction. A deputation is now being sent to the Lord Chancellor and the Home Secretary to press on them the views of the Association.

The most important matter which came up for discussion at the representative meeting was the question how to meet the coming Poor Law Reform and Government Insurance against Illness. The latter alone is to embrace 15,000,000 of wage-earners, perhaps the greatest revolution with which the profession has ever been faced.

Will this Association, which includes a majority of the medical men in the country, be able to make a united stand, and properly present our side of the case to Parliament? If individual voices crying alone for this or that, and totally unorganised, are to be heard, then it is perfectly certain that the needs and the just claims of the general practitioner will be put on one side. The general public will neither understand nor listen to the grievances of individuals, but an Association of 21,000 members can and will make its voice heard if well supported.

The Committee on Poor Law Reform has already drawn up an elaborate scheme, which has gone through a preliminary discussion by the Council and the Representative meeting, and will soon be sent to the divisions, with alternative plans and supplementary reports on the working of the German system, and possibly a report from an actuary. After the replies of the divisions have been digested a special Representative meeting, probably in February, will decide finally on the lines to be adopted. The Chancellor of the Exchequer has expressed his willingness to receive deputations, and the Friendly Societies are arranging to lay their case before him. Here then is a unique opportunity for every medical man in the country to join in moulding the proposals of a united profession, and to say to the Government: If you wish to purchase our services, these and these only are the conditions under which we can deal with you.

The scheme at present contemplates a central national provident medical service, organised and controlled by the Association, though without any financial liability. It will be a federation or union of local services, each controlled by (1) a medical committee organised by the local branch of the Association, with representatives of non-members, and by (2) a mixed committee of laymen and medical men for matters referred to them. There will be a direct contract between each medical officer and his patient, or the person or body who pays for him, but the terms of the contract, such as the rate of fees, must first be agreed upon by the medical committee. The benefits of the service will be restricted to those within certain wage limits; contributions will be received from public bodies, employers and others. Any doctor will have the right to refuse a patient, and the patient will have his choice among all the doctors on the list. The payments will be put into a local fund from which the medical officers will be paid as may be agreed on, either at so much a head for each patient or in proportion to the attendance actually given. Some of us would favour a plan under which private practitioners would simply send in accounts at ordinary rates for attendance on Government beneficiaries.

The attractions of London itself are so great, and the special attractions provided for the annual meeting of 1910 in the Metropolis rendered it likely that as regards the number attending the records of previous years should be far exceeded. Everything appears to have been so well arranged that all the details worked themselves out with *éclat*, and the Metropolitan Counties Branch well deserves the congratulations of all who attended the most brilliantly successful meeting in the history of the Association.

At Westminster Abbey there was a large and varied array of colours due to the academic costumes of many Universities, a large number of the wearers of these robes being ladies. The sermon by the Very Rev. W. Page Roberts, Dean of Salisbury, was one which will not soon be forgotten by those who were present. The question, "Does it matter what a man believes?" was the subject of a powerful discourse, in which the doctrines of Nietsche were contrasted with the Christian faith, the preacher contending that the wild beast morality of Nietsche, if adopted, would not only supplant the morality of Christ, but would also supplant the medical man's mission of mercy.

The addresses of the President (Henry T. Butlin, D.C.L., LL.D), that on "Medicine," by Dr. J. Mitchell Bruce, LL.D., and that on "Surgery" by Gilbert Barling, F.R.C.S., were all worthy of an occasion when the respective speakers have the opportunity of a lifetime to say to a large concourse of their fellow practitioners what may be burning within them. It was, however, unfortunate that the lecturer on "Medicine" had to address his audience in a room which had little oxygen left, and in which at the commencement of the lecture there was only standing room at the top where nothing could be heard.

It was a fortunate occurrence that the Right Hon. Lord Ilkeston (Sir Walter Foster) was able to attend Mr. Butlin's dinner at the Hotel Cecil, to be congratulated on his elevation to the peerage, and that His Excellency the Earl of Aberdeen was present to do honour to the British Medical Association by attendance at the annual dinner. Those who attended the meeting at Montreal will not have forgotten all that was then done by Lord Aberdeen, in his capacity of Governor of the Dominion of Canada, when he attended all the public meetings, and spoke on many occasions words of welcome of the Association to Canada.

The various social entertainments were most enjoyable. The Guildhall reception was on the usual scale which characterises the hospitality of the Corporation of London; the guests were received by Sir John Knill and the Lady Mayoress in the historic hall, and many entertainments of an enjoyable character were provided. The garden party at Ranelagh on a fine afternoon, with its very interesting polo match, was much appreciated.

Various other meetings and the Saturday excursions have all been more or less described in the *British Medical Journal*, and need no further comment. We must, however, say a word about the medical museum, which is well described as "transcending all previous efforts in this direction." The museum committee had made a unique collection, drawn from all the medical schools and other museums and laboratories of London, and carefully arranged into various sections, each under the care of an expert honorary curator. An ocular demonstration of recent advances, and of the methods employed both in the practice and in the laboratory for the diagnosis and treatment of disease, gave a marvellous revelation to most of the visitors of the great advances in tropical medicine, in parasitology and bacteriology which have been made in recent years. A collection of three hundred microscopes, lent by Messrs. E. Leitz, materially enhanced the utility of this exhibition.

The annual exhibition of foods, drugs, etc., was of the usual character, and contained much of novelty and interest.

**Conference on Tuberculosis, Edinburgh.**

MUCH has been done by the National Association for the Prevention of Consumption and other forms of tuberculosis since its inauguration by the late King Edward VII some ten years ago. The mortality from tuberculosis has shown a great diminution, and more particularly so in consequence of the establishment of sanatoria in nearly all parts of the country. In our own district various local centres were formed, many meetings were held, and a branch of the Association was founded in each of the three adjoining counties of Gloucester, Somerset and Wilts. Funds were collected over the area of these counties, including the city of Bristol, and after soms delay it was decided to at once take proceedings for the establishment of a three counties sanatorium, which was to be located in a spot conveniently accessible from all parts of this area. The spot chosen was on the hills overlooking Limpley Stoke, above the Avon Valley, in the Parish of Winsley, and the sanatorium established there has become familiar to us under the name of the Winsley Sanatorium.

Founded in 1904, it has had five years of useful activity, and the fifth annual report for the year 1909 gives good evidence that it has become an indispensable part of the work of combating tuberculosis in this district. Having accomplished this much, the various local committees appear to have fallen asleep, and to have been content wtih what they had done. The President of the three counties branch was appointed President of the

Sanatorium Board of Management, and we were fortunate in having the cordial assistance of Lord Islington, formerly Sir John Dickson-Poynder, Bart., who having now become Governor of New Zealand, has of necessity resigned the office to which he was appointed at the foundation of the Sanatorium. The appointment of a successor will now rest with the Board of Management of the Sanatorium; but meanwhile the National Association is still alive, as is shown by the very successful annual meeting recently held in Edinburgh, on July 4th, under the presidency of Lord Balfour of Burleigh, Chairman of the Council.

The subject which attracted the greatest attention was the incidence of tuberculosis in childhood, when the views of Dr. Hamburger, of Vienna, were discussed, and threw some doubt on the value of the tuberculin test for diagnosis. The statement of Dr. Hamburger that "nearly all persons in the city of Vienna at the age of fourteen years were already tuberculous" appeared to be almost incredible, but the frequency of tuberculosis in children appears not yet to be fully appreciated. Dr. R. W. Philip believed that tuberculosis was essentially a disease begun in childhood; it was the tuberculosis of the child that made the tuberculosis of the adult in the great majority of cases. Hence the necessity for special provision for the children who are found to be tuberculous, and for the manifestation of care in this respect in the carrying out of the work of medical inspection of the school children. We trust that the National Association of Great Britain will continue its crusade in conjunction with the various similar organisations of other countries, and that their efforts will be as successful in the future as they have been during the past decade.

**Dr. John Brown.**

THIS year marks the centenary of the birth of the author of *Rab and His Friends*. Dr. John Brown was born at Biggar, a small town in Lanarkshire, on September 22nd, 1810, the son of a distinguished Presbyterian divine. He practised medicine for nearly fifty years in Edinburgh, where he and the dogs who shared his carriage on his rounds were well-known figures.

Although his practice was never very large, and as a scientist he did not attract popular applause, medicine owes to his memory a heavy debt; his contributions to the humanities of our profession still retain their force. He was one of a small group of doctors who, while continuing in active practice, have made their mark as men of letters. Our national literature has always been rich in essayists, and his essays rank with Lamb's, and with Lamb's alone in our language. As one of his biographers has said, "Writing of nothing that he did not know, he wrote, too, of nothing that he did not love." This is the secret of the high level at which he kept his art.

In his writings of and to children he has no equal. His story of Pet Marjorie stands by itself. Oliver Wendell Holmes, in one of his letters to Dr. Brown, said, "Most of all have I read, and re-read, and then insisted on reading for the third time aloud to my wife, that infinitely tearful, smileful, soulful, tender, caressing—where shall I stop?—story of Pet Marjorie. Dear little soul."

His humour is quaint and rich. We like few things better than the tale of the shoemaker explaining all his miseries to his minister, finishing up with, "And besides the sweep ower the street has bocht my hoose, and is gaun tae pit me oot." This climax of woe the minister tried to meet in various ways, but in vain, so on leaving he advised Jeems to "*lay it before the Lord.*" In a fortnight the minister called and found Jeems quite cheery, whistling away on his *Crepida.* "Ye 're better, I see, James; how is that?" "Oh, sir, *I did as you bade me, and the sweep 's deed!*"

His *Notes on Art* bring vividly back to mind the spirit which inspired and appreciated the early Victorian painters. We who now scorn their pictures and execrate their art may almost applaud the efforts which won his approval in such terms. And his taste is not even now wholly rejected. Raeburn has come to rank as a portrait painter nearly where John Brown placed him, and we would not have one word of his *Memoir of Leech* erased or rewritten. We can learn from him, better even than from his friend Ruskin, how to explain, if not to excuse, the master-painters of British art in those days.

It is strange in reading their letters to find how much Ruskin, for all his infallibility, relied upon the encouragement, sympathy and good sense of his "best and truest friend," and how far too he fell short of Dr. Brown in receptiveness and breadth of view. Ruskin realised, enviously perhaps, the wider range of John Brown's sympathies in art. "How did you get to understand Beethoven?" he wrote. "He always sounds to me like the upsetting of bags of nails, with here and there an also dropped hammer." In *Hallé's Recital* is concentrated a whole-hearted joy in music, untrammelled it may be by technical knowledge, but large and wide enough to touch life at many points. One reads few such criticisms of music in these modern times. How he must have loved that of which he wrote, "Who can forget Mozart's 'Jupiter,' or the overture to *Der Freischütz,* or the delicious, innocent, Elysian 'Surprise' of Haydn—as if it had wandered out of Paradise, or the many-voiced sonatas of Beethoven—deep, mobile, unfathomable, melancholy as the sea?"

The best of his writings for us, however, are those which deal with the practice and profession of medicine, *Locke and Sydenham, Our Gideon Grays, Art and Science, Education through the Senses,* and *Happy Guessing.* Here we see him battling hard to blend the diverse factors of pure science and applied medicine, laying straight hold on the middle propositions which lie between exact facts and speculative science, and have to do with immediate application and act. It is a fine contrast that he draws between the art and the science in medicine, where he says the former "is *ante-mortem,* and runs for the stomach pump;" while the latter "is *post-mortem,* and studies the phenomena of poisoning."

But his own standpoint is well illustrated in his preface to the 1866 edition of *Horæ Subsecivæ.* "I am almost ashamed of slipping into this volume the rambling paper on 'Vaughan,' my only excuse, and it is none, being that the gentle and heavenly-minded Silurist was a country surgeon. Perhaps a better excuse would be that I like to show that our medicus may be not only, like Locke, at once a good physician and metaphysician, or like

Adams, equally great as a scholar and a domestic 'leech,' but that he may be a poet too; and, moreover, that we hard-worked family doctors, when the day's work is over, and our books posted, our letters answered, and our newspapers duly studied, may take up our Tennyson, our Wordsworth, our Dryden, our Cowper, our Shakespeare, or our Scott, and read ourselves pleasantly asleep in our arm-chair."

Medicine is the richer for such men as John Brown, who lived, practised and wrote as the advocate for going back to the old manly, intellectual and literary culture of the days of Sydenham and Arbuthnot, Heberden and Gregory; when a physician fed, enlarged, and quickened his entire nature; when he lived in the world of letters as a freeholder, and reverenced the ancients, while at the same time he pushed on among his fellows and lived in the present.

He died as he had lived, feeling the full delight—

"That when our days do end, they are not done,
And though we die, we shall not perish quite,
But live two lives where others have but one."

